# Abnormal Static and Dynamic Functional Connectivity in Left and Right Temporal Lobe Epilepsy

**DOI:** 10.3389/fnins.2021.820641

**Published:** 2022-01-20

**Authors:** Xiaomin Pang, Xiulin Liang, Jingyuan Zhao, Peirong Wu, Xinrong Li, Wutong Wei, Liluo Nie, Weiwei Chang, Zongxia Lv, Jinou Zheng

**Affiliations:** Department of Neurology, The First Affiliated Hospital of Guangxi Medical University, Nanning, China

**Keywords:** temporal lobe epilepsy, functional magnetic resonance imaging, static functional connectivity, dynamic functional connectivity, cognitive deficits

## Abstract

**Objective:**

Temporal lobe epilepsy (TLE) can be conceptualized as a network disease. However, the network characteristics in lateralization remain controversial.

**Methods:**

In this study, resting-state functional MRI scans were acquired from 53 TLE patients [22 with left-side TLE (LTLE) and 31 with right-side TLE (RTLE)] and 37 matched healthy controls. We focused on the characteristics of static and dynamic functional connectivity, including static connectivity patterns and topological properties, as well as temporal properties of the dynamic connectivity state and the variability of the dynamic connectivity and network topological organization. Correlation analyses were conducted between abnormal static and dynamic properties and cognitive performances.

**Results:**

The static functional connectivity analysis presented a significantly decreased cortical-cortical connectivity pattern and increased subcortical-cortical connectivity pattern in RTLE. The global-level network in RTLE showed a significant decrease in global efficiency. The dynamic functional connectivity analysis revealed that RTLE patients exhibited aberrant connectivity states, as well as increased variability in the subcortical-cortical connectivity. The global-level network in RTLE revealed increased variance in global efficiency and local efficiency. The static or dynamic functional connectivity in LTLE did not show any significant abnormalities. The altered dynamic properties were associated with worsening cognitive performance in language and conceptual thinking by the TLE patients.

**Conclusion:**

Our findings demonstrated the presence of abnormalities in the static and dynamic functional connectivity of TLE patients. RTLE patients exhibited more pronounced aberrant connectivity patterns and topological properties, which might represent a mechanism for reconfiguration of brain networks in RTLE patients. These observations extended our understanding of the pathophysiological network mechanisms of TLE.

## Introduction

Temporal lobe epilepsy (TLE) is the most common type of localization-related epilepsy associated with recurrent spontaneous seizures and widespread cognitive dysfunction. Over the past decades, compelling evidence from semeiology, histopathology, electrophysiology, and neuroimaging has revealed that the neurobiological insults in TLE extend beyond the epileptogenic temporal lobe into extratemporal regions, supporting the concept that TLE is a disorder of large neural networks (Spencer, 2002; Jiruska et al., 2014). The connectome analysis, as applied to data from structural metrics or neurophysiologic signals, allows assessment of the aberrant connectivity between different regions in the brain network, as well as the network topological features related to integration and segregation, which has helped develop the network hypothesis of TLE (Bernhardt et al., 2013; Cataldi et al., 2013).

Due to the hemispheric asymmetries of the human brain, the different network organization patterns involved in left-side TLE (LTLE) and right-side TLE (RTLE) have long been recognized. Based on correlations observed among cortical and subcortical volumes, structural covariance network analysis conducted by Yasuda et al. (2015) demonstrated reduced cortical/subcortical connectivity and a less than optimal topological organization in TLE patients compared to controls. Although both LTLE and RTLE patients displayed decreased global efficiency, increased local efficiency, and an increased clustering coefficient, more severe abnormalities were observed in LTLE. A global white matter connectome analysis also described a more pronounced reduction in connectivity in LTLE than RLTE (Besson et al., 2014). This pattern of more pronounced structural network alterations is consistent with the more severe morphological abnormalities seen in gray and white matter in LTLE. However, not all studies have reported a similar lateralization effect for the structural network pattern. One recent structural network study indicated that the structural disruptions of networks in patients with LTLE and RTLE were differed in distribution and severity; RTLE exhibited more extensive abnormalities than patients with LTLE (Yu et al., 2019). Lemkaddem et al. (2014) found that patients with RTLE experienced more severe connectivity alterations at the whole-brain, hub, and regional levels. The laterality of the epileptogenic zone also exerts a distinct impact on the functional network derived from resting-state functional magnetic resonance imaging (fMRI) data. In published findings, the functional network of RTLE and LTLE are different, and the network pattern is as controversial as the structural network. As reported by Vytvarova et al. (2017), a significant disturbance in functional connectivity was observed only in LTLE; the global functional network showed a higher clustering coefficient and characteristic path length. Chiang et al. (2014) identified more pronounced reductions in the functional connectivity of the limbic network in RTLE. The tendency toward reorganization in topological changes in LTLE was thought to help explain lateralized variations in neurobehavior and cognition. It is unclear whether the network abnormalities in TLE arise from pathologic mechanisms directly related to seizure impact or whether they are an adaptive mechanism to prevent the loss of functional integrity. The heterogeneous hemispheric lateralization effect also has not been clearly explained. Thus, further evidence is needed concerning the network pathology of TLE, especially for functional networks.

In most previous studies, it has been assumed that the functional interactions remained constant during fMRI scanning. In that context, functional connectivity represents an average across complex spatio-temporal phenomena. Recent studies highlight the abundant information contained within the dynamic characteristics of functional connectivity (Hutchison et al., 2013; Cohen, 2018; Liégeois et al., 2019). Dynamic functional connectivity has been applied to characterize the inherent dynamic properties of brain networks in clinical populations, including schizophrenia (Lemkaddem et al., 2014), Parkinson's disease (Chiang et al., 2014; Vytvarova et al., 2017), and Alzheimer's disease (Liégeois et al., 2019). This information has led to a better understanding of the underlying neural mechanisms of the disorders that cannot be discovered through static functional connectivity alone. For patients with TLE, only a few studies have focused on the fluctuations of connectivity within predefined regions of interest, such as the hippocampus (Morgan et al., 2020). The characterizations of dynamic functional connectivity in TLE from the perspective of global networks remain largely unknown.

In this study, we investigated the differences in static and dynamic functional connectivity patterns among healthy controls as well as LTLE and RTLE patients. Graph theory methods were employed to capture the global properties that reflected the information flow in the brain networks. Correlation analyses also were carried out between abnormal characteristics and cognitive performances. We expected to gain new insight into the physiological networks affected by lateralization.

## Materials and Methods

### Participants

Fifty-three TLE patients with unilateral TLE [including left-side TLE (LTLE) and right-side TLE (RTLE)] were enrolled through the Epilepsy Clinic of the First Affiliated Hospital of Guangxi Medical University from December 2018 to January 2020. Diagnosis and localization of TLE were determined by comprehensive evaluation, including a detailed history and seizure semiology, ictal/interictal scalp electroencephalography, and routine clinical MRI according to the guidelines of the International League Against Epilepsy (ILAE) (Berg et al., 2010; Scheffer et al., 2017). The inclusion criteria were as follows: (1) the patients were right-handed; (2) the typical clinical semiology was consistent with seizures of temporal lobe origin; (3) ictal/interictal scalp electroencephalograms demonstrated epileptic discharges originating from a unilateral temporal lobe; and (4) the patients had been regularly taking antiepileptic drugs (AEDs). The exclusion criteria included the existence of clinical or electrographic evidence of bitemporal or extratemporal seizures, developmental anomalies, cortical malformations, or other focal lesions on the clinical MRI, a history of severe mental or neurological disease other than epilepsy, any history of substance or alcohol abuse, inability to complete all of the procedures in this experiment, and contraindications for MRI. A subset of the patients exhibited MRI evidence of hippocampal atrophy, a common finding in TLE. However, this was not used as an inclusion or exclusion criterion.

Thirty-seven healthy control (HC) subjects without a history of mental or neurological disease were recruited from the surrounding community. They were matched to the patient groups with respect to age, gender, and years of education. This study was approved by the Medical Research Ethics Committee of the First Affiliated Hospital of Guangxi Medical University. Written informed consent was obtained from each subject.

### Cognitive Data

The Montreal Cognitive Assessment (MoCA) (Nasreddine et al., 2005), a brief cognitive screening tool, was administered to all participants. Several researchers have suggested using the MoCA to screen patients with epilepsy (Phabphal and Kanjanasatien, 2011; Yang et al., 2018). We utilized the Chinese version of the MoCA Basic, which was translated from the original English with subtle linguistic and cultural modifications (Chen et al., 2016). This screening tool assesses several cognitive domains, including executive function, language, orientation, calculation, conceptual thinking, memory, visuoperception, naming, attention, and concentration.

### MRI Data Acquisition

All participants underwent structural and functional data acquisition using an Achieva 3.0 T MRI system scanner (Philips, Amsterdam, The Netherlands) with a standard eight-channel head coil. Earplugs were used to reduce scanner noise. Foam padding was placed between the patient's head and coil to minimize head movements. Participants were instructed to remain awake and relaxed, avoid thinking about any specific topic, and keep their eyes closed during the scanning. T1-weighted high-resolution data were acquired with the turbo field echo sequence, using the following parameters: repetition time = 7.8 ms, echo time = 3.4 ms, flip angle = 9°, field of view = 256 × 256 mm, matrix size = 256 × 256, number of slices = 176 with no gap, voxel size = 1 × 1 × 1 mm. Functional MRI data were obtained using an echo-planar imaging sequence: repetition time = 2,000 ms, echo time = 30 ms, flip angle = 90°, field of view = 220 × 220 mm, matrix size = 64 × 62, number of slices = 41, slice gap =0.5 mm, and voxel size = 3.44 × 3.44 × 3.5 mm. The total time for the functional MRI acquisition was 7 min 30 s, and 225 volumes were collected.

### MRI Data Pre-processing

Pre-processing of functional data was performed using the Data Processing and Analysis for Brain Imaging toolbox (DPABI V4.2, www.rfmri.org/dpabi), which is based on Statistical Parametric Mapping software (SPM12, www.fil.ion.ucl.ac.uk/spm). The first ten volumes were removed in the functional data pipeline to allow for signal stabilization. The remaining volumes underwent slice timing correction and realignment. To eliminate the influence of head motion, participants were excluded if they presented mean frame-wise displacement (FD) values > 0.2 mm, or if the maximum displacement was > 3 mm, or 3° in angular rotation (Power et al., 2014; Yin et al., 2021). The functional images were co-registered to the individual T1 image, then normalized to the Montreal Neurological Institute (MNI) space using an affine transformation with the voxels resampled to a resolution of 3 × 3 × 3 mm. Finally, the normalized images were spatially smoothed using a 6 mm full-width at half-maximum Gaussian kernel.

### Group Independent Component Analysis

After pre-processing, the functional data were decomposed into functional networks using the group independent component analysis ICA of fMRI Toolbox (GIFT v4.0b, http://icatb.sourceforge.net/). The pipeline of the spatial group ICA basically followed the steps described in the classic article by Allen et al. (2014). A two-step data reduction was used to reduce the functional data dimension. For subject-specific data reduction, a total of 120 principal components across each subject were retained using the standard economy-size decomposition algorithm. In the group-level data reduction, the concatenated subject-reduced data were decomposed into 100 group independent components using the expectation maximization algorithm. The Infomax ICA algorithm was run 20 times using ICASSO to estimate the reduction stability. Independent components with average intra-cluster similarity values > 0.8 were selected. Then, the spatial maps and time courses were back-reconstructed for each subject.

The meaningful independent components were identified based on the criteria recommended by Allen et al. (2011). Briefly, the spatial map of components exhibited peak activations in gray matter and showed low spatial overlap with known vascular, ventricular, and susceptibility artifacts and edge regions corresponding to head motion. Also, the spectral powers of the corresponding time courses were dominated by low-frequency fluctuations, and the low-frequency to high-frequency power ratio was higher than 4. In the current study, 43 independent components were ultimately retained.

### Static and Dynamic Functional Connectivity Network Construction

Before evaluating functional connectivity, the time courses for the 43 independent components were further processed to detrend, despike, and filter using a high-frequency cutoff of 0.15 Hz (Allen et al., 2011). A 43 × 43 functional connectivity matrix was created for each subject by performing Pearson's correlations for time courses of pairwise independent components. The static functional connectivity was defined as a measure of average connectivity during the entire scan duration, while the dynamic functional connectivity was computed using the sliding-window approach. In line with previous studies (Allen et al., 2014; Fiorenzato et al., 2019), the time courses were divided into windows of 22 TRs with a Gaussian, σ = 3 TRs. The onset of each window progressively slid in steps of one TR from the previous one, resulting in 193 windows. This segment length has been demonstrated to provide a good compromise between the quality of the correlation matrix estimation and the temporal resolution. For each subject, the resulting 193 functional connectivity matrixes represented the dynamic changes of functional connectivity during the resting state scan period. The values for the static and dynamic functional connectivity matrixes were transformed to z-scores to improve normality.

### Static Functional Connectivity Analysis

#### Static Connectivity Analysis

Connectivity analysis was performed on the static functional connectivity matrix. The MANCOVAN utility within GIFT was used to test the effect of group membership on functional network connectivity. The design matrix included group membership (HC, LTLE, or RTLE) as a covariate of interest. Age, sex, education level, and mean FD were considered nuisance covariates. Multivariate analysis of covariance was conducted to identify factors that influenced the response matrix, and then univariate tests were performed with a reduced design matrix. Only group membership showed a significant relationship with functional network connectivity. Therefore, univariate tests were performed using a term for group membership. The significance criterion was *P* < 0.05 with false discovery rate (FDR) correction for multiple comparisons.

#### Static Graph Theory Analysis

A graph theory method was applied to examine the topological organization of the static functional connectivity networks. The topological properties on the static functional connectivity matrixes across all subjects were calculated using the graph theoretical network analysis toolbox (GRETNA v2.0, www.nitrc.org/projects/gretna). Each functional connectivity matrix was binarized with respect to a set of fixed sparsity thresholds. The threshold range of sparsity was identified as 0.08–0.48 in 0.01 increments, based on a previous study (Song et al., 2021). Only positive relationships were used in the analysis. The small-world properties (including normalized clustering coefficient, normalized characteristic path length, and small-worldness index) and network efficiency (including global network efficiency and local network efficiency) were examined at each sparsity threshold. To avoid the specific selection of a threshold, we applied an area under the curve (AUC) approach, which is widely used in graph theory-based network studies (Tu et al., 2019). The AUC changes for each topological property were calculated for statistical comparisons. Age, sex, education level, and mean FD were considered nuisance covariates.

### Dynamic Functional Connectivity Analysis

#### Connectivity State Analysis

The variability in functional brain connectivity gave rise to highly structured patterns of connectivity that emerged and dissolved over time, which are called connectivity states (Allen et al., 2014; Yu et al., 2015). To explore the frequency (measured by temporal occurrence) and structure (measured by representing strength and directionality) of recurring functional connectivity patterns over time, the k-means clustering algorithm was applied to cluster the states of the windowed functional connectivity matrixes across all subjects. We used the squared Euclidean distance method for the k-means in this study, and 500 iterates and 150 replicate functional connectivity windows were partitioned into different clusters. The optimal number of clusters was determined based on the silhouette criterion of the cluster validity index (Fiorenzato et al., 2019). The temporal properties of the functional connectivity state, including the reoccurrence fraction and mean dwell time in each state, as well as the total transition number between different states, were calculated for each subject.

#### Connectivity Variability Analysis

To assess the variability in individual functional connectivity across sliding windows, the variance of the 193 windowed functional connectivity matrixes mentioned above was calculated for statistical comparisons. Age, sex, education level, and mean FD were considered nuisance covariates. The significance criterion was *P* < 0.05, with false discovery rate (FDR) corrections for multiple comparisons. A larger variance indicated more variable (or less stable) functional connectivity of the networks.

#### Dynamic Graph Theory Analysis

A graph theory method also was applied to examine the variability of the topological organization of the dynamic functional connectivity networks across windows. Similar to the static graph theory analysis, calculations of the topological properties on the 193 functional connectivity matrixes across all subjects were performed using the GRETNA toolbox. The variance of the AUC changes for each topological property was calculated for statistical comparisons. Age, sex, education level, and mean FD were considered nuisance covariates.

### Statistical Analysis

The statistical analyses were conducted in SPSS v. 22.0. The one-sample Kolmogorov-Smirnov test was used as the normal distribution test for quantitative data. The variables of the demographics data demonstrated normal distribution. Therefore, one-way ANOVAs (three levels: HC, LTLE, and RTLE) were used to determine the significance, and *post-hoc* analysis was conducted if a significant difference was found. Differences in clinical characteristics between the LTLE and RTLE groups were detected using a two-sample *t*-test (continuous data) or a chi-square test (categorical data) as appropriate. The variables of the MoCA scores and dynamic properties exhibited non-normal distribution, so the Kruskal-Wallis test was used to compare cognitive profiles across groups. *Post-hoc* analysis was used to determine from which groups the differences originated. Partial correlation analyses were conducted to detect associations between the MoCA performance and the altered dynamic properties. Age, sex, education level, and mean FD were treated as controlling covariates. The significance criterion was *P* < 0.05, and the Bonferroni correction was applied for multiple comparisons.

## Results

### Demographics and Clinical Characteristics

**Figure 1:**
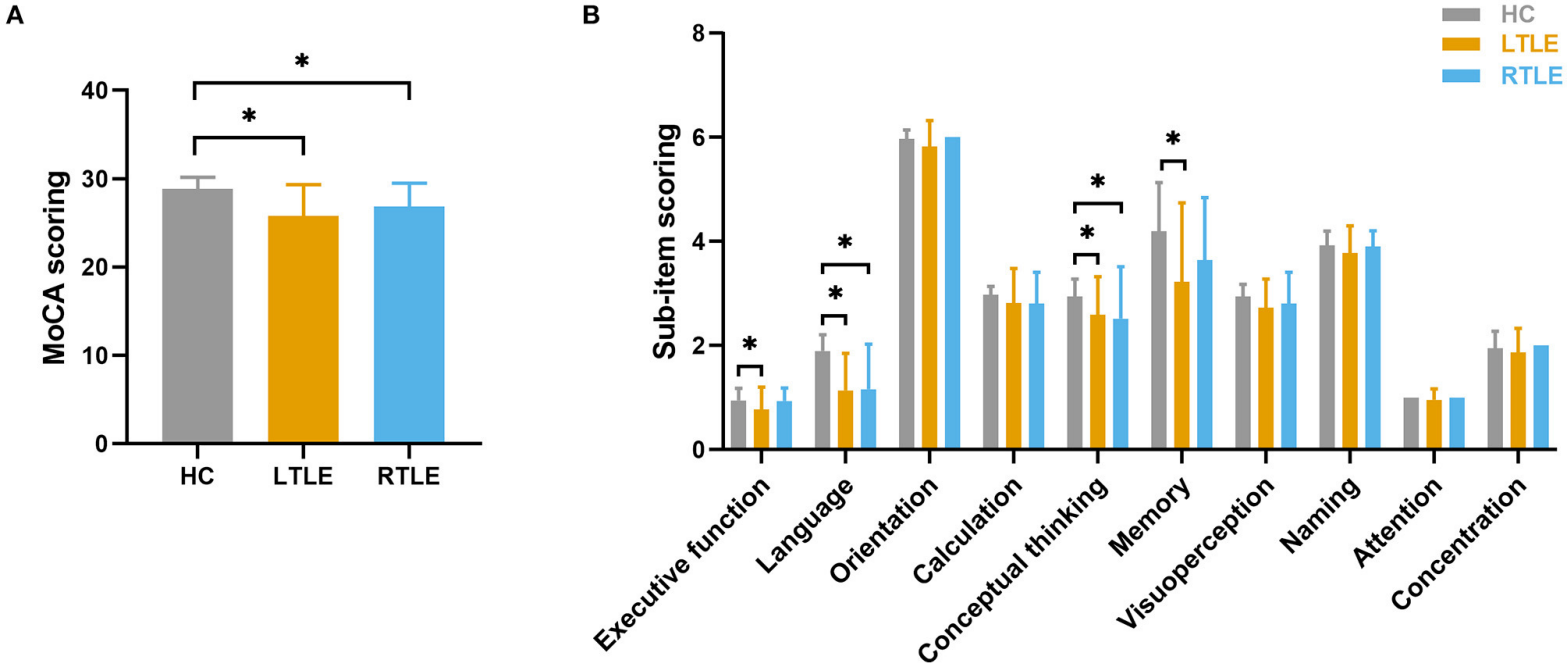
Group differences in cognitive performance as assessed by the MoCA. **(A)** Group comparison in MoCA total scores. **(B)** Group comparison using sub-item scores of MoCA. The horizontal lines above the bars indicate comparisons that achieved statistically significant thresholds (*P < 0.05, Bonferroni corrected).

Fifty-three TLE patients (22 with LTLE and 31 with RTLE) and 37 controls were included in the study. The demographic data and clinical features of all subjects are summarized in Table 1. No significant differences were observed among the three groups with regard to age, sex, and education level. No significant differences were found in the epilepsy-related features between patients in the LTLE and RTLE groups, including age at onset of epilepsy, epilepsy duration, seizure frequency, seizure type and the number of current AEDs taken. Significant differences were observed in cognitive performance assessed with MoCA among the three groups, as shown in Figure 1. Patients with LTLE primarily exhibited impairments in executive function, language, conceptual thinking, and memory. Patients with RTLE exhibited impairments in language and conceptual thinking.

**Table 1:** Demographic characteristics and clinical features of patients with TLE and healthy controls.

	**HC** **(***n*** = 37)**	**TLE**		**F/χ^2^/t**	***P*** **-value**
		**LTLE** **(***n*** = 22)**	**RTLE** **(***n*** = 31)**		
**Demographic characteristics**					
Age (years)	29.57 ± 7.74	29.95 ± 8.10	31.42 ± 9.00	0.447	0.641^a^
Gender (male/female)	16/21	6/16	8/23	2.789	0.248^b^
Education level (years)	13.38 ± 2.34	13.31 ± 2.77	13.45 ± 2.47	0.019	0.981^a^
**Clinical features**					
Age at onset (years)	NA	21.57 ± 10.17	21.05 ± 10.25	0.182	0.856^c^
Epilepsy duration (years)	NA	8.39 ± 6.13	10.34 ± 5.53	−1.211	0.231^c^
Seizure frequency (time/months)	NA	4.91 ± 9.16	2.50 ± 3.86	1.313	0.195^c^
Seizure type (Focal/FBTCS)	NA	8/14	15/16	0.757	0.384^b^
AEDs (mono-/polytherapy)	NA	10/12	17/14	0.453	0.501^b^

*Values are presented as means ± standard deviation. For all tests, P < 0.05 was considered statistically significant*.

a *P-values were obtained by one-way ANOVA*.

b *P-values were obtained by the chi-square test*.

c *P-values were obtained by a two-sample t-test*.

*HC, healthy control; LTLE, left-side TLE; RTLE, right-side TLE; FBTCS, focal to bilateral tonic–clonic seizure*.

### Intrinsic Connectivity Networks Identification

**Figure 2:**
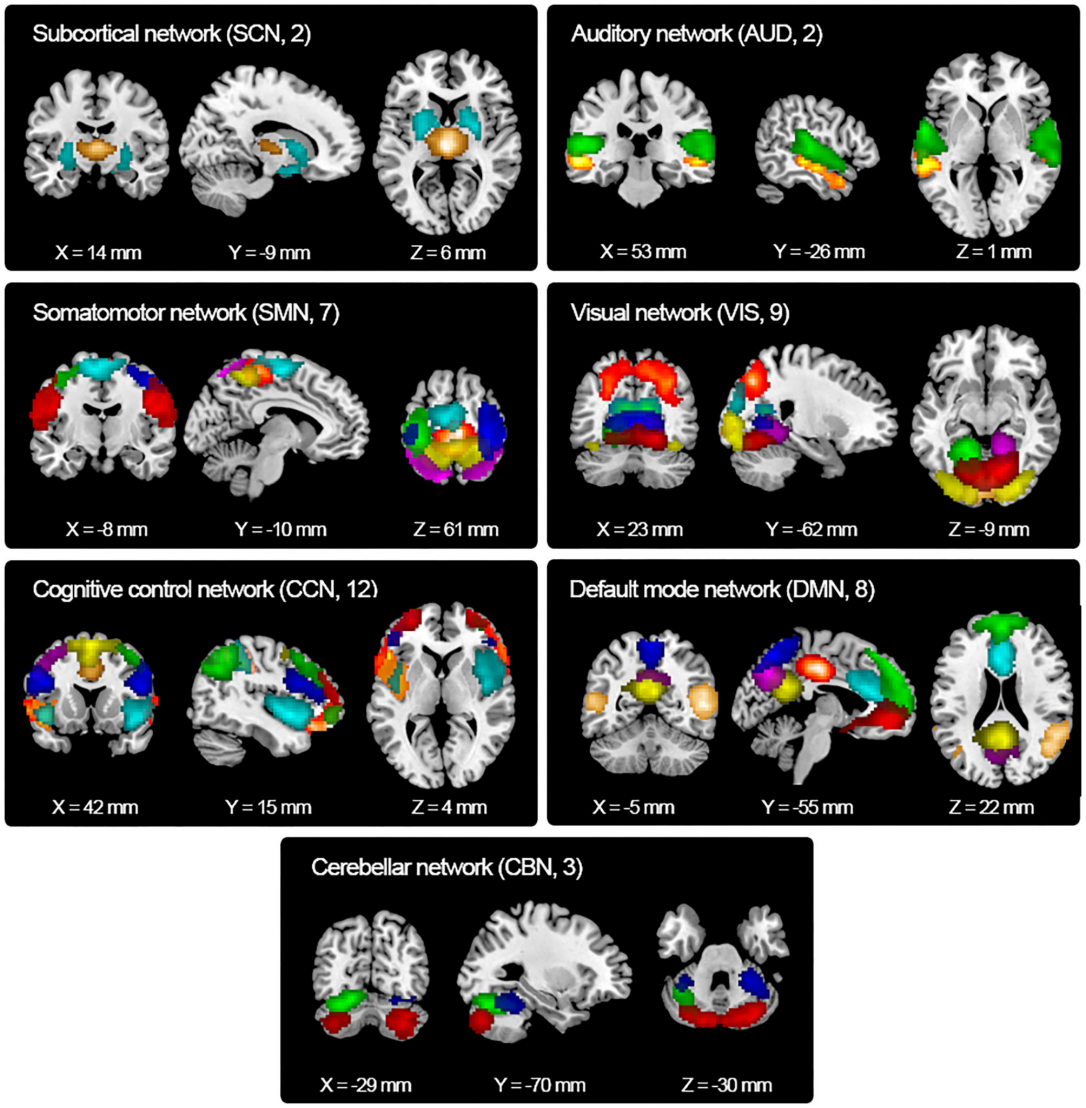
Spatial maps of the identified independent components. Forty-three independent components were identified and sorted into seven functional networks based on their anatomical and functional properties. SCN, subcortical network; AUD, auditory network; SMN, somatomotor network; VIS, visual network; CCN, cognitive control network; DMN, default mode network; CBN, cerebellar network.

Resting-state fMRI data were decomposed into functional independent components using group spatial ICA. Spatial maps of 43 independent components were identified and are shown in Figure 2. Based on the anatomical and presumed functional properties, these independent components were arranged into seven functional networks, including a subcortical network (SCN), auditory network (AUD), somatomotor network (SMN), visual network (VIS), cognitive control network (CCN), default mode network (DMN), and cerebellar network (CBN). Detailed images of each independent component are shown in Supplementary Figure 1, and the coordinates for the peak activations are listed in Supplementary Table 1.

### Static Functional Connectivity Analysis

#### Static Connectivity Patterns

**Figure 3:**
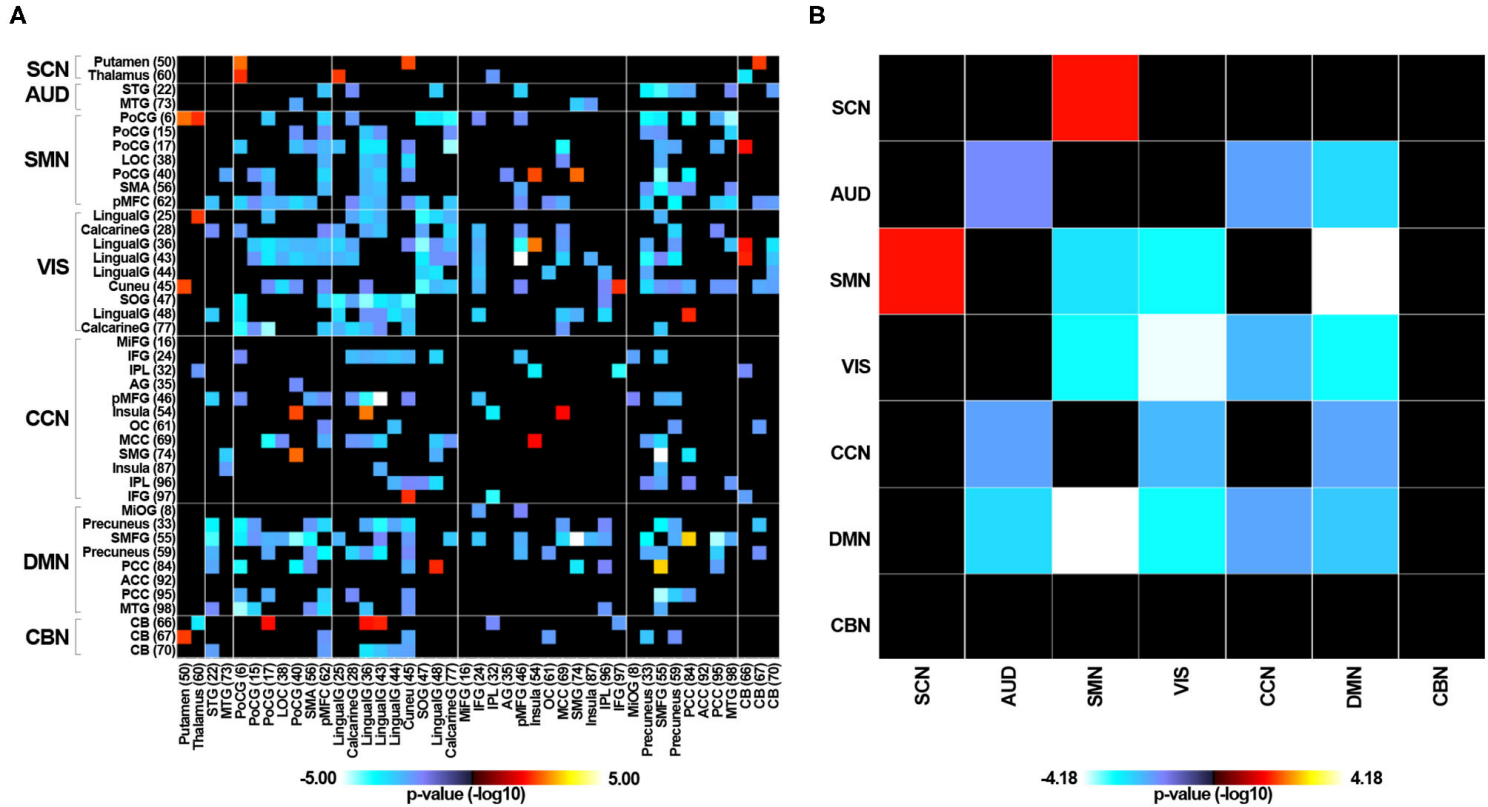
The group comparisons of static functional connectivity. **(A)** Group difference of static connectivity of 43 independent components between the RTLE and HC groups (*P* < 0.05, FDR corrected). **(B)** Group difference of static connectivity of seven functional networks between the RTLE and HC groups (*P* < 0.05, FDR corrected).

Supplementary Figure 2 displays the averaged static functional connectivity network between the independent components computed over the entire scan. The static connectivity within the AUD, SMN, VIS, and DMN networks, as well as between the SCN and SMN, the AUD and CCN, the AUD and DMN, the VIS and SMN, the VIS and CCN, the VIS and DMN, and the CCN and DMN networks showed significant differences among the three groups (*P* < 0.05, FDR corrected). Specifically, compared with the HC group, the RTLE group exhibited significantly decreased connectivity within the AUD, SMN, VIS, and DMN networks, as well as decreased connectivity between the AUD and CCN, the AUD and DMN, the VIS and SMN, the VIS and CCN, the VIS and DMN, and the CCN and DMN networks. Comparison between the HC and RTLE groups also revealed increased connectivity between the SCN and SMN networks, shown in Figure 3. There was no difference between the LTLE and HC and RTLE and LTLE groups.

#### Static Graph Theory Analysis

**Figure 4:**
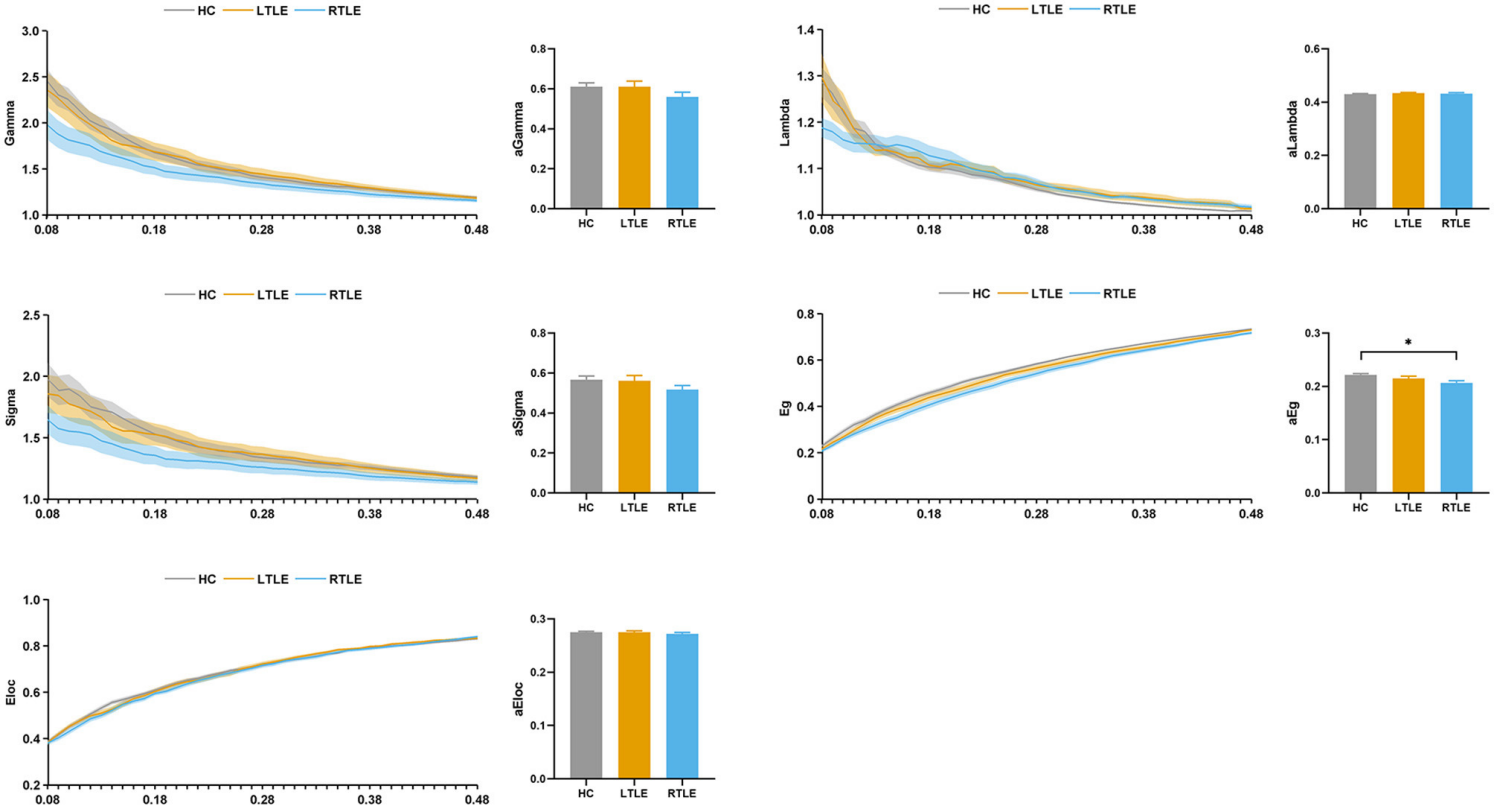
Global topological properties in static functional connectivity. The left column represents the small-world properties and network efficiency across the sparsity range 0.08–0.48. Straight lines show the average, and the corresponding transparent areas indicate the standard error. The right bar demonstrates the group effect of AUC. The horizontal lines above the bars indicate comparisons that achieved statistically significant thresholds (*P < 0.05, Bonferroni corrected). Gamma, normalized clustering coefficient; Lambda, normalized characteristic path length; Sigma, small-worldness index; Eg, global network efficiency; Eloc, local network efficiency.

The topologic properties of the static connectivity network were calculated and compared among the groups. As seen in Figure 4, the global network efficiency showed significant differences among the three groups. The RTLE group exhibited lower global network efficiency compared with the HC group. There was no difference in the global network efficiency between the LTLE and HC and the RTLE and LTLE groups. There were no differences in other global properties, i.e., the normalized clustering coefficient, normalized characteristic path length, small-worldness index, and local network efficiency among the three groups.

### Dynamic Functional Connectivity Analysis

#### Connectivity State Analysis

**Figure 5:**
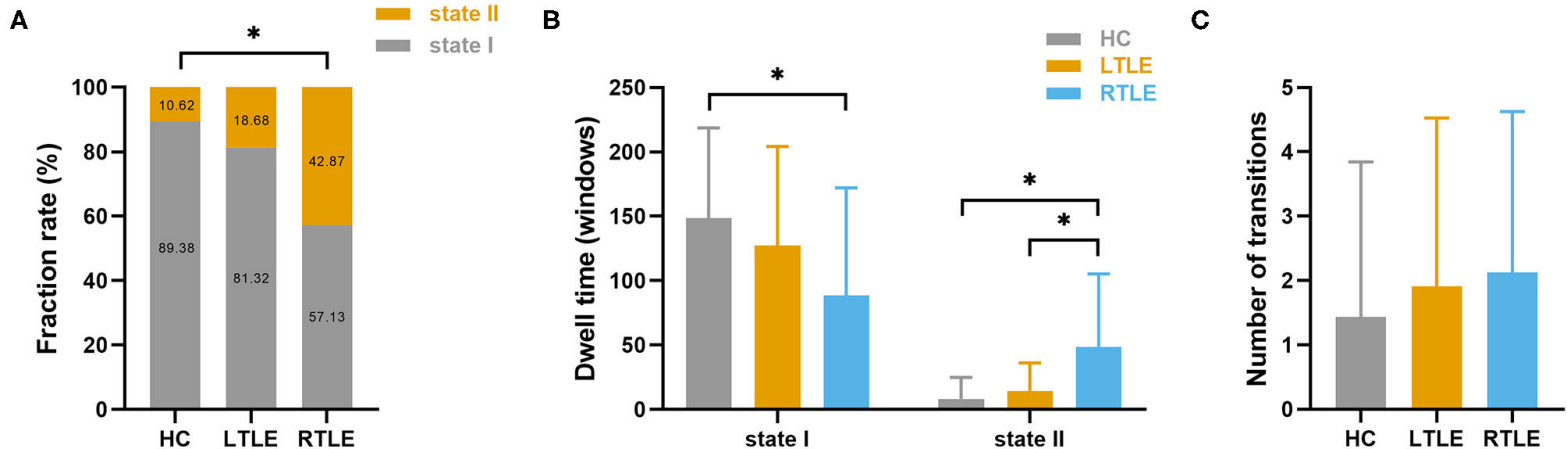
Temporal properties and connectivity of the dynamic functional connectivity state. Group differences in the fraction rate **(A)**, mean dwell time **(B)**, and the number of transitions of each state **(C)** were measured. The horizontal lines above the bars indicate comparisons that achieved statistically significant thresholds (*P < 0.05, Bonferroni corrected).

Clustering analysis revealed two connectivity states that recurred throughout the individual scans and across subjects. Supplementary Figure 3 shows the centroids of the clusters for each state, which reflected a connectivity pattern that was stably present within the data. Significant group differences were detected in the fraction rate of occurrences and the mean dwell time for each state, as seen in Figure 5. Specifically, compared with the HC group, the RTLE group exhibited a significantly lower fraction rate in state I and a higher fraction rate in state II. The mean dwell time for the RTLE group in state I was significantly shorter compared with the HC group, and the mean dwell time for the RTLE group in state II was significantly longer than the HC and LTLE groups. No differences were observed in the number of transitions between groups.

#### Connectivity Variability Analysis

**Figure 6:**
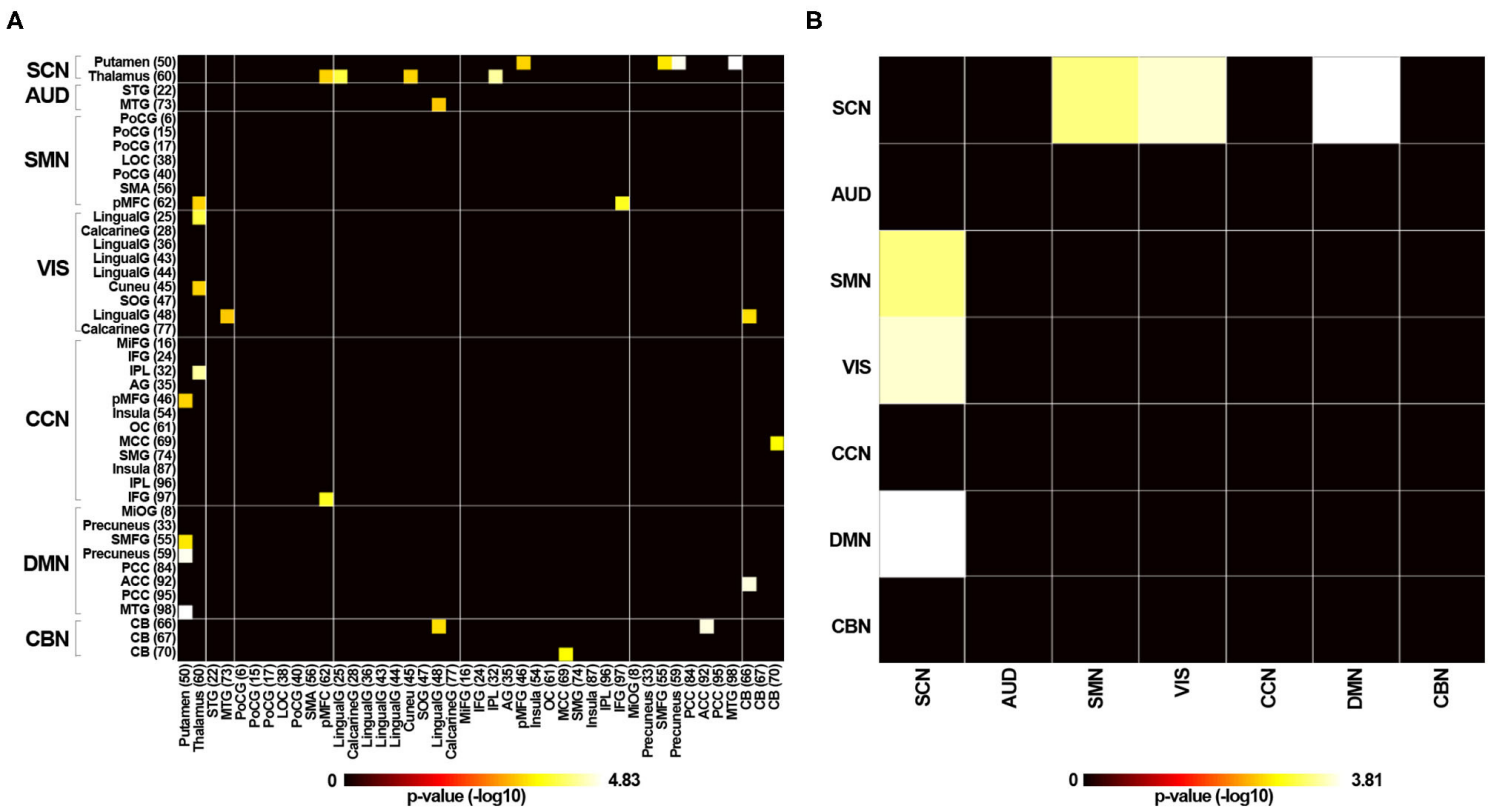
Variability in dynamic functional connectivity across windows. **(A)** Group difference of connectivity variability of 43 independent components between the RTLE and HC groups (*P* < 0.05, FDR corrected). **(B)** Group difference of connectivity variability of seven functional networks between the RTLE and HC groups (*P* < 0.05, FDR corrected).

We estimated the temporal variability in the dynamic functional connectivity using the variance in functional connectivity across sliding windows. The connectivity variability between the SCN and SMN, the SCN and VIS, and the SCN and DMN networks showed significant differences among the three groups (*P* < 0.05, FDR corrected). Specifically, compared with the HC group, RTLE patients exhibited significantly increased connectivity variability between the SCN and SMN networks, the SCN and VIS networks, and the SCN and DMN networks, as seen in Figure 6. There were no differences between the LTLE and HC and RTLE and LTLE groups.

#### Dynamic Graph Theory Analysis

**Figure 7:**
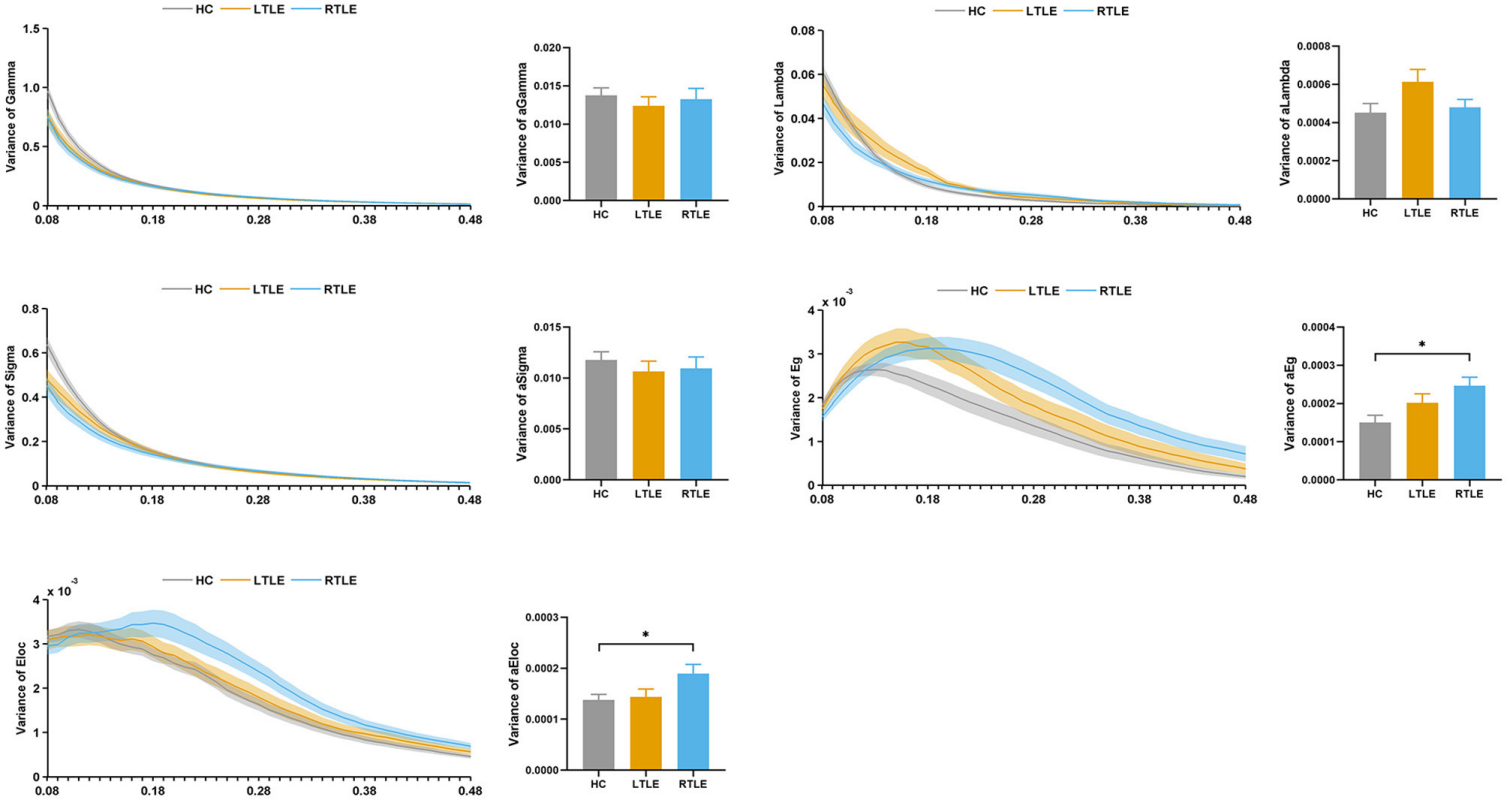
Variability of global topological properties in dynamic functional connectivity. The left column represents the variance of small-world properties and network efficiency for the sparsity range 0.08–0.48. Straight lines show the average, and the corresponding transparent areas indicate the standard error. The right bar demonstrates the group effect of AUC. The horizontal lines above the bars indicate comparisons that achieved statistically significant thresholds (*P < 0.05, Bonferroni corrected). Gamma, normalized clustering coefficient; Lambda, normalized characteristic path length; Sigma, small-worldness index; Eg, global network efficiency; Eloc, local network efficiency.

The variability of the graph metrics with respect to dynamic changes across windows was calculated and compared among the groups. As demonstrated in Figure 7, the variance of the global network efficiency and local network efficiency showed significant differences among the three groups. The RTLE group exhibited increased variance in the global network efficiency and local network efficiency compared with the HC group. There were no differences in the variance of the global network efficiency and local network efficiency between the LTLE and HC and RTLE and LTLE groups. There were no differences in the variance of normalized clustering coefficient, normalized characteristic path length, and small-worldness index among the three groups.

### Correlation Between Dynamic Properties and MoCA Scores

**Figure 8:**
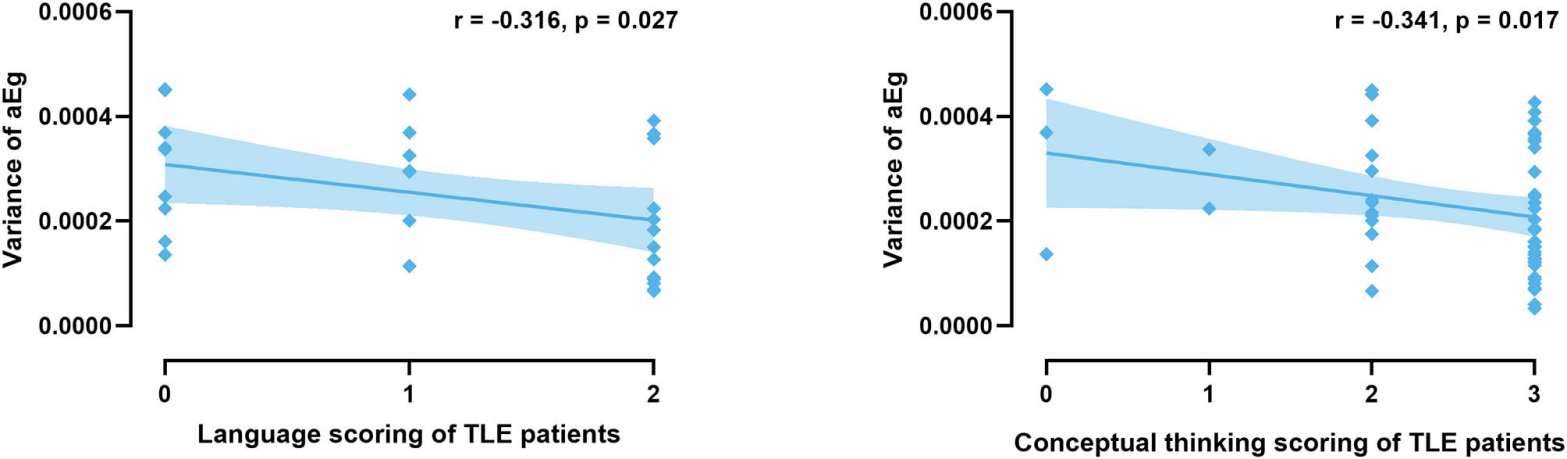
Significant correlations are present between characteristics and cognitive performance in all TLE patients.

Correlation analyses were performed to test whether functional connectivity characteristics were associated with cognitive performance, as seen in Figure 8. We observed that the variance in the global network efficiency was negatively correlated with cognitive performance for language and conceptual thinking (*P* < 0.05, uncorrected).

## Discussion

Complex changes in functional connectivity affected by lateralization in TLE are far more complicated than we currently understand. In the present study, we focused on the static and dynamic connectivity measures between unilateral TLE patients and healthy individuals. Several findings emerged. (1) The static functional connectivity analysis presented a significantly decreased cortical-cortical connectivity and increased subcortical-cortical connectivity patterns in RTLE. The global-level network in RTLE showed a significant decrease in global network efficiency. However, the static functional connectivity in LTLE did not display any significant abnormalities. (2) The RTLE patients exhibited aberrant connectivity states and increased variability in the subcortical-cortical connectivity. The global-level network in RTLE showed increased variance in the global network efficiency and local network efficiency. However, the dynamic functional connectivity in LTLE did not manifest significant abnormalities. (3) The altered dynamic properties were associated with worsening cognitive performance by the TLE patients in language and conceptual thinking. These observations extended our understanding of the brain network disorders in unilateral TLE patients.

In the past decade, characterizing the functional connectivity patterns has become a powerful tool to explore the epileptic brain. Our findings concerning the static functional connectivity were similar to previous findings (Zhang et al., 2009; Maneshi et al., 2014). The results revealed significantly decreased connectivity within DMN, perceptual networks (i.e., VIS, AUD, and SMN), as well as the connectivity between DMN and perceptual networks in RTLE patients. Decreased connectivity between CCN and perceptual networks also was observed. The decreased functional connectivity resulted from disruptions in neuronal connections and reflected functional impairments associated with brain disorders. The perceptual networks are responsible for information communication with the external environment. The DMN and CCN play complementary roles in supporting cognitive control, allowing the individual to navigate multiple streams of brain information flexibly. These disrupted intra-network and inter-network connectivity could explain the hallmark pathological functions, including seizure semiology, as well as behavioral and cognitive deficits. On the other hand, increased functional connectivity is often interpreted to reflect enhanced functionality due to a compensatory mechanism. The increased functional connectivity between subcortical structures and the regions from SMN, VIS, or DMN systems observed in the current study probably resulted from a compensatory response in the decreased cortical-cortical connectivity in TLE. These enhanced patterns of subcortical-cortical connection highlighted that the subcortical structures also play an essential role in the disordered network in TLE.

Our study also extended results from static functional connectivity analysis to a more subtle time scale. Using sliding windows and k-means clustering, we identified two connectivity patterns that recurred throughout the entire scanning time. State I accounted for the largest percentage of windows and time and was primarily characterized by positive couplings located primarily within distinct networks (i.e., SMN, VIS, and DMN). In contrast, state II occurred less frequently and presented stronger positive couplings between networks (i.e., SMN-VIS, DMN-SMN, DMN-VIS, and DMN-CBN). In the RTLE group, the reoccurrence fraction and dwell time of the within-network connected state (state I) was decreased compared to the HC group, while the between-network pattern (state II) increased. This unstable state underscored the vulnerability of the functional network in TLE. Specifically, RTLE patients presented less frequency in the within-network connected state, which supported the concept of reduced functional segregation of functional networks. The RTLE patients spent more time in the between-network connected state, which could be interpreted as a potential compensatory mechanism to regain cortical homeostasis in an abnormal brain. Although LTLE and RTLE patients did not experience more state transitions than HC participants, we observed a significant increase in the temporal variability of functional connectivity across the sliding windows.

Zhang et al. (2016) characterized a stable brain-wide topography of functional connectivity variability in healthy subjects, indicating the degree of flexibility and adaptability in various brain regions. They found that regions showing extreme variability (either highest or lowest) in healthy subjects, such as the DMN and subcortical areas, also showed significant disease-specific variability changes in mental disorders. Similar observations were reported in patients with generalized tonic-clonic seizures (GTCS). Compared to healthy controls, GTCS patients revealed increased variability of functional connectivity in cognition-related networks, especially in the DMN. This alteration in connectivity variability was relatively consistent across different methods and templates, which reflected a dynamic restructuring of the brain network (Jia et al., 2020). One study of TLE patients focused on the instability index of connectivity within the DMN using the non-overlapping time windows approach. This study revealed that two region pairs, the precuneus-left inferior parietal and the precuneus-left middle temporal, exhibited higher levels of unstable connections compared to controls. Also, this instability was more pronounced for RTLE patients and associated with a longer disease duration (Robinson et al., 2017). In the current study, the subcortical structures appeared to be the region that consistently expressed higher variability in their relationships with other regions. Higher variability in connectivity was observed between the putamen or thalamus and the SMN, VIS, or DMN systems. Ample evidence has demonstrated that the subcortical nuclei, especially the thalamus, amplify the spread of seizures and play crucial roles in regulating broad epileptogenic networks in TLE (Guye et al., 2006). Atrophy of the thalamus coexistent with the temporal lobe possibly reflects neuronal loss secondary to seizures (Barron et al., 2012). Furthermore, damage to the thalamocortical network, particularly ipsilateral to the epileptogenic focus, was observed in our previous study (Chen et al., 2015). The results of the high variability analysis that we observed here extend static network research to the temporal dynamic domain. Given that the thalamus is increasingly well-described as a gatekeeper to control the information interaction across cortical networks, we believe that dysfunction in the thalamocortical functional connectivity is a core neurobiological abnormality in TLE.

The brain systems in adults exhibit efficient information segregation and integration at a cost-efficiency balance (Liao et al., 2017). Graph theory has the potential to illuminate the topological characteristics related to global integration and local segregation characteristics of complex functional networks at the whole-brain level. Thus, both the static and dynamic analyses included graph theory measures in the current study. The differences in global topological properties of functional connectivity between TLE patients and healthy individuals have been reported in numerous studies. In our present static functional connectivity results, the global network efficiency was significantly decreased in RTLE patients compared to HCs, suggesting a lower efficiency of parallel information transfer. Higher variability in dynamic functional connectivity also transcended local connections. The global network efficiency, which measures the network's ability for information transfer, exhibited increased variance in RTLE patients, implying less efficient and more unstable information transfer within the functional network and suggested that abnormalities existed in the global network integration. Increased variance in the local network efficiency, a measure of the efficiency with which a given node communicates with the rest of the brain, was observed in RTLE patients, suggesting poor stationarity of the local segregation. In contrast, the small-world index among TLE patients consistently exhibited temporal stationarity, indicating that an optimal balance existed between global integration and local segregation. These results confirmed and extended the findings of static functional connectivity described above.

In this study, we demonstrated the presences of abnormal static and dynamic functional connectivity in TLE patients relative to controls, including the functional connectivity patterns and network topological properties. It is notable that TLE showed a different pattern of functional connectivity in terms of lateralization. In published findings, the functional connectivity network characteristics in RTLE and LTLE remain variable and in conflict. These observations might be affected by sample size, MRI collection parameters, analysis methods, and others. Dynamic functional connectivity could provide more information concerning the inherent characteristics of the networks that cannot be discovered through static functional connectivity alone. We applied both static and dynamic functional connectivity approaches in the current study and observed that patients with RTLE exhibited greater alterations than patients with LTLE. The differences between the RTLE and HC groups were significant after multiple comparison corrections, but the LTLE and HC groups were not different. It appears that the cognitive dysfunction in TLE patients also exhibited a lateralization effect, with RTLE tending to be less severely affected. Patients with LTLE demonstrated more severe deficits in executive function, language, conceptual thinking, and memory. Conversely, patients with RTLE exhibited impairments only in language and conceptual thinking. Also, the observed increased variance in global network efficiency was negatively associated with cognitive performance. Epilepsies are considered dynamic diseases of brain systems in which neuronal networks change between a normal mode of activity and a seizure mode (Da Silva et al., 2003; Richardson, 2012). Given that the brain is a remarkably adaptive and plastic organ (Pedersen et al., 2017), abnormal dynamic network properties might reflect a restructuring of the brain networks that respond and adapt to epileptic activity, protecting the epileptic brain from a continuously ictal state. Thus, we speculate that the significant abnormal dynamic connectivity in RTLE might serve to stabilize neural networks and act as an adaptive mechanism to prevent the loss of functional integrity in the epileptic brain.

Several limitations of this study need to be considered. First, all patients included in this study were taking AEDs. Previous studies have reported on the effects of AEDs on functional networks (Pang et al., 2020). We agree that it is challenging to dissociate the relative contribution of AEDs from the observations noted in this study. Thus, it is critical to distinguish AED-related effects on dynamic functional connectivity properties by comparing these results with drug-naïve patients in the future. Second, directed effective connectivity among brain regions was introduced recently to explain the connectivity dynamics in the brain (Park et al., 2018; Zarghami and Friston, 2020). The functional connectivity network we constructed in this study did not provide information on the directionality or causality of the connections. An attractive approach in TLE patients in the future would be to generate effective connectivity data features that change over time. This would provide an essential step toward a better understanding of functional connectivity affected by lateralization in TLE.

## Conclusions

Overall, these observations documented abnormalities in static and dynamic functional connectivity in TLE patients. We found that RTLE was associated with more pronounced aberrant connectivity patterns and topological properties, which might be a compensatory mechanism subsequent to epileptic activity. These findings extended our understanding of the pathophysiological network in TLE.

## Data Availability Statement

The original contributions presented in the study are included in the article/Supplementary Material, further inquiries can be directed to the corresponding author.

## Ethics Statement

This study was approved by the Medical Research Ethics Committee of the First Affiliated Hospital of Guangxi Medical University. Written informed consent to participate in this study was provided by the participants' legal guardian/next of kin.

## Author Contributions

JZ contributed to conception and design of the study. XP and XL wrote the first draft of the manuscript. JZ and PW wrote sections of the manuscript. XL, WW, and LN organized the data collection. WC and ZL performed the statistical analysis. All authors contributed to manuscript revision, read, and approved the submitted version.

## Funding

This study was supported by a grant from the National Natural Science Foundation of China (Nos. 81560223, 81660225) and Natural Science Foundation of Guangxi Province (2021GXNSFBA220031).

## Conflict of Interest

The authors declare that the research was conducted in the absence of any commercial or financial relationships that could be construed as a potential conflict of interest.

## Publisher's Note

All claims expressed in this article are solely those of the authors and do not necessarily represent those of their affiliated organizations, or those of the publisher, the editors and the reviewers. Any product that may be evaluated in this article, or claim that may be made by its manufacturer, is not guaranteed or endorsed by the publisher.
