# Supplementary material for: Abnormal Static and Dynamic Functional Connectivity in Left and Right Temporal Lobe Epilepsy
Source: Front Neurosci. 2022 Jan 20;15:820641. doi: 10.3389/fnins.2021.820641 (PMC8813030; doi:10.3389/fnins.2021.820641)

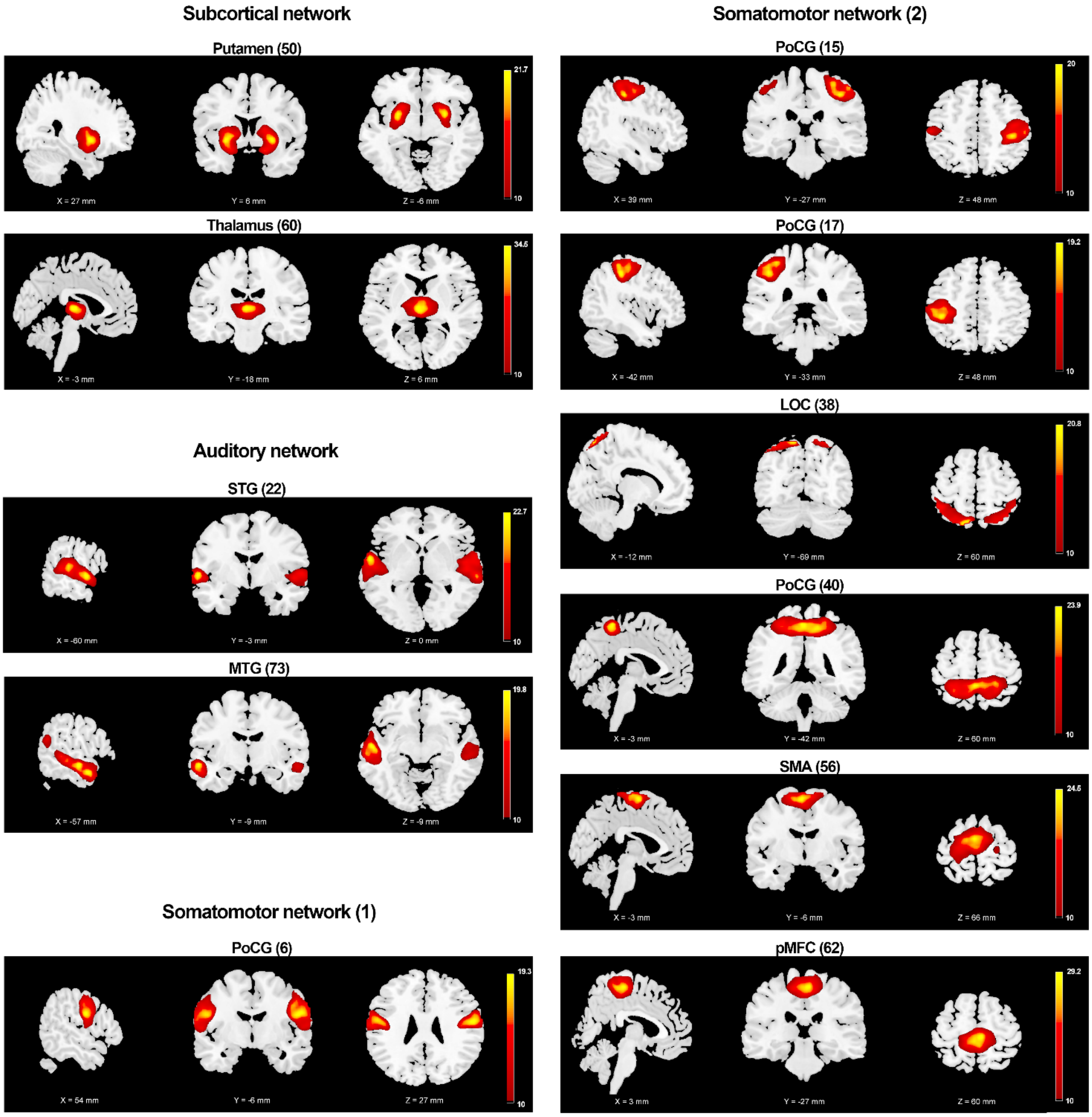

Supplementary Figure 1. Spatial Maps of independent component. Each independent component is thresholded at |t| > 10, where one-sample t-statistics have been computed across the entire subject group. The MNI coordinates of the peak values for each spatial map are displayed below the three orthogonal planes. The color bar represents t values.

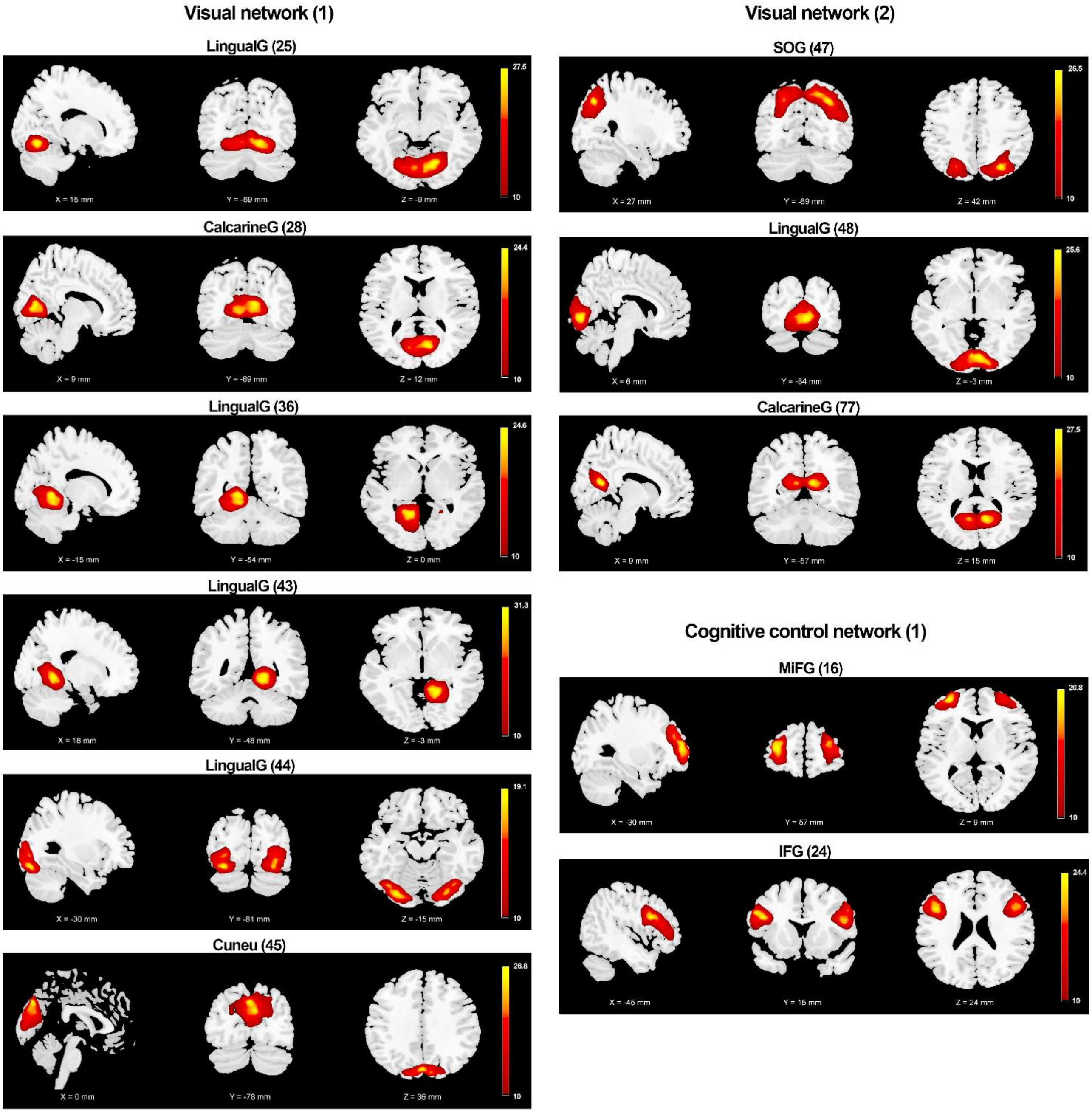

Visual network (1)
LingualG (25)
X = 15 mm
Y = -89 mm
Z = -9 mm
CalcarineG (28)
X = 9 mm
Y = -69 mm
Z = 12 mm
LingualG (36)
X = -15 mm
Y = -54 mm
Z = 0 mm
LingualG (43)
X = 18 mm
Y = -48 mm
Z = -3 mm
LingualG (44)
X = -30 mm
Y = -81 mm
Z = -15 mm
Cuneu (45)
X = 0 mm
Y = -78 mm
Z = 36 mm
Visual network (2)
SOG (47)
X = 27 mm
Y = -69 mm
Z = 42 mm
LingualG (48)
X = 6 mm
Y = -84 mm
Z = -3 mm
CalcarineG (77)
X = 9 mm
Y = -57 mm
Z = 15 mm
Cognitive control network (1)
MiFG (16)
X = -30 mm
Y = 57 mm
Z = 9 mm
IFG (24)
X = -45 mm
Y = 15 mm
Z = 24 mm

Cognitive control network (2)

IPL (32)

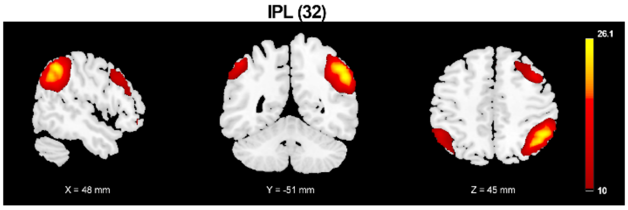

AG (35)

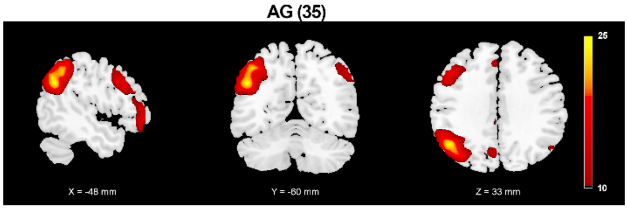

pMFG (46)

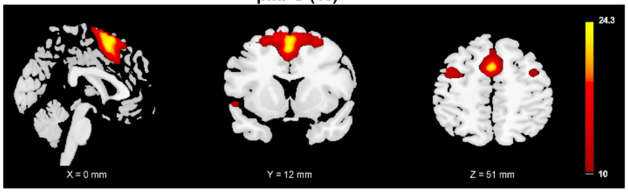

Insula (54)

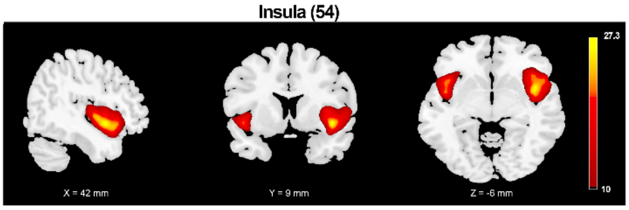

OC (61)

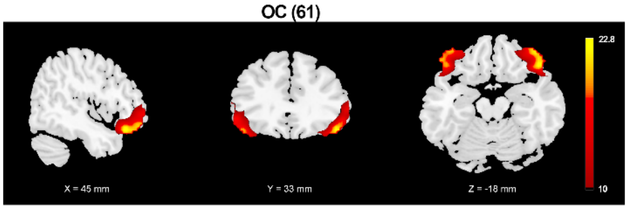

MCC (69)

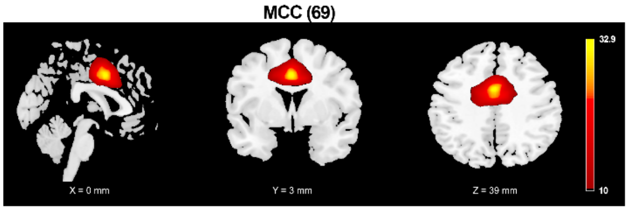

Cognitive control network (3)

SMG (74)

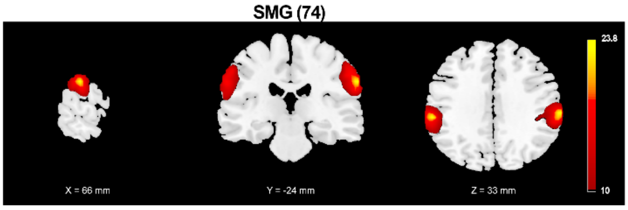

Insula (87)

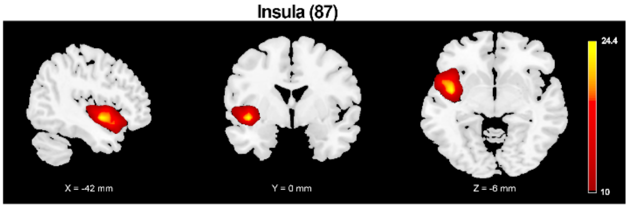

IPL (96)

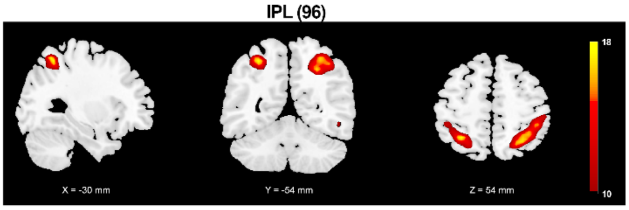

IFG (97)

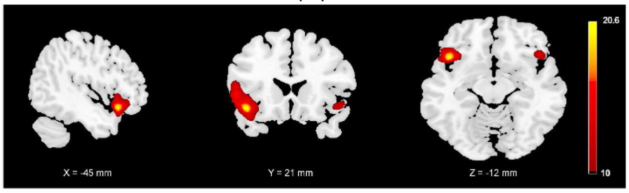

Default mode network (1)

MiOG (8)

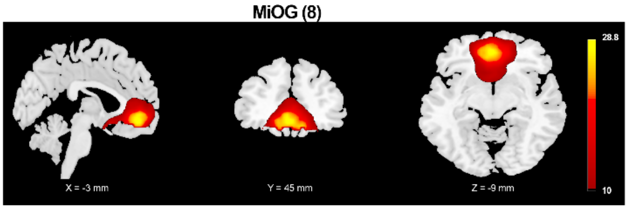

Default mode network (2)

Precuneus (33)

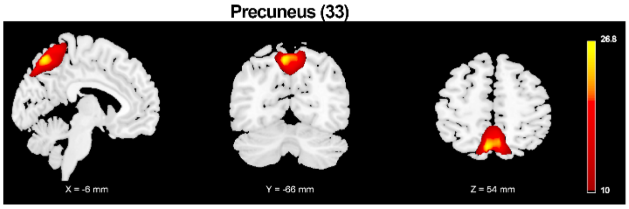

SMFG (55)

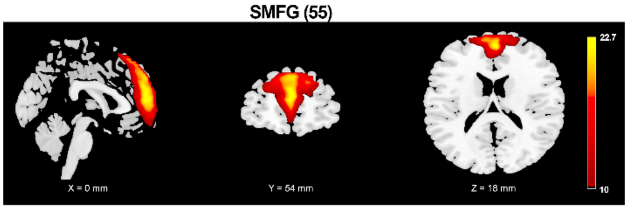

Precuneus (59)

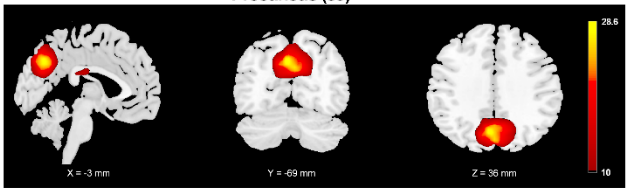

PCC (84)

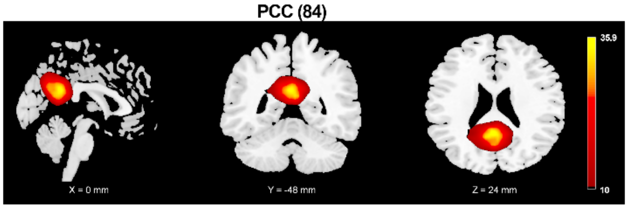

ACC (92)

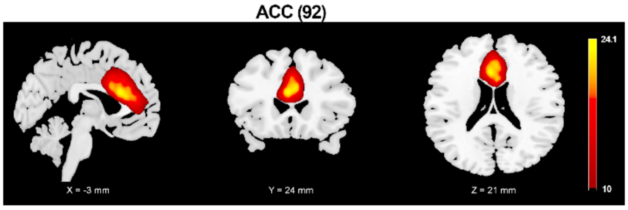

PCC (95)

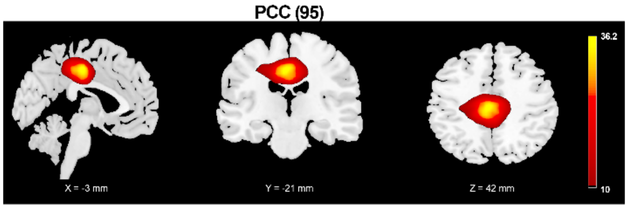

Default mode network (3)

MTG (98)

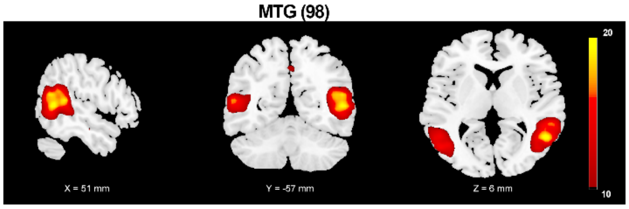

Cerebellar network

CB (66)

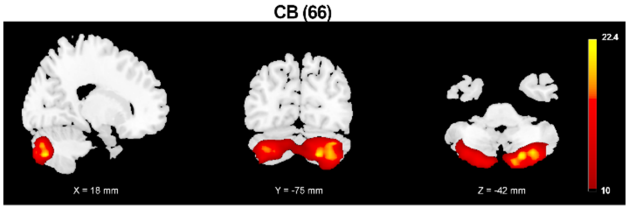

CB (67)

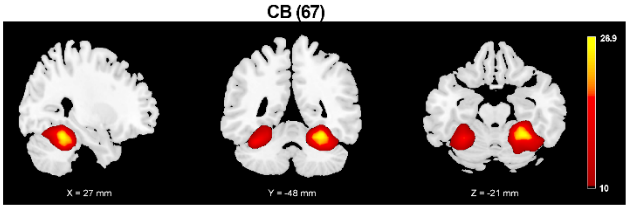

CB (70)

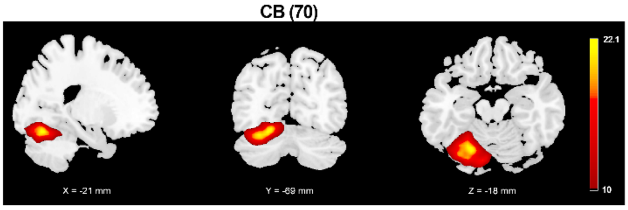

Supplement: Supplementary file 1 [file Image_1.pdf]
