# Supplementary material for: Abnormal Static and Dynamic Functional Connectivity in Left and Right Temporal Lobe Epilepsy
Source: Front Neurosci. 2022 Jan 20;15:820641. doi: 10.3389/fnins.2021.820641 (PMC8813030; doi:10.3389/fnins.2021.820641)
Supplement: Supplementary file 2 [file Image_2.pdf]

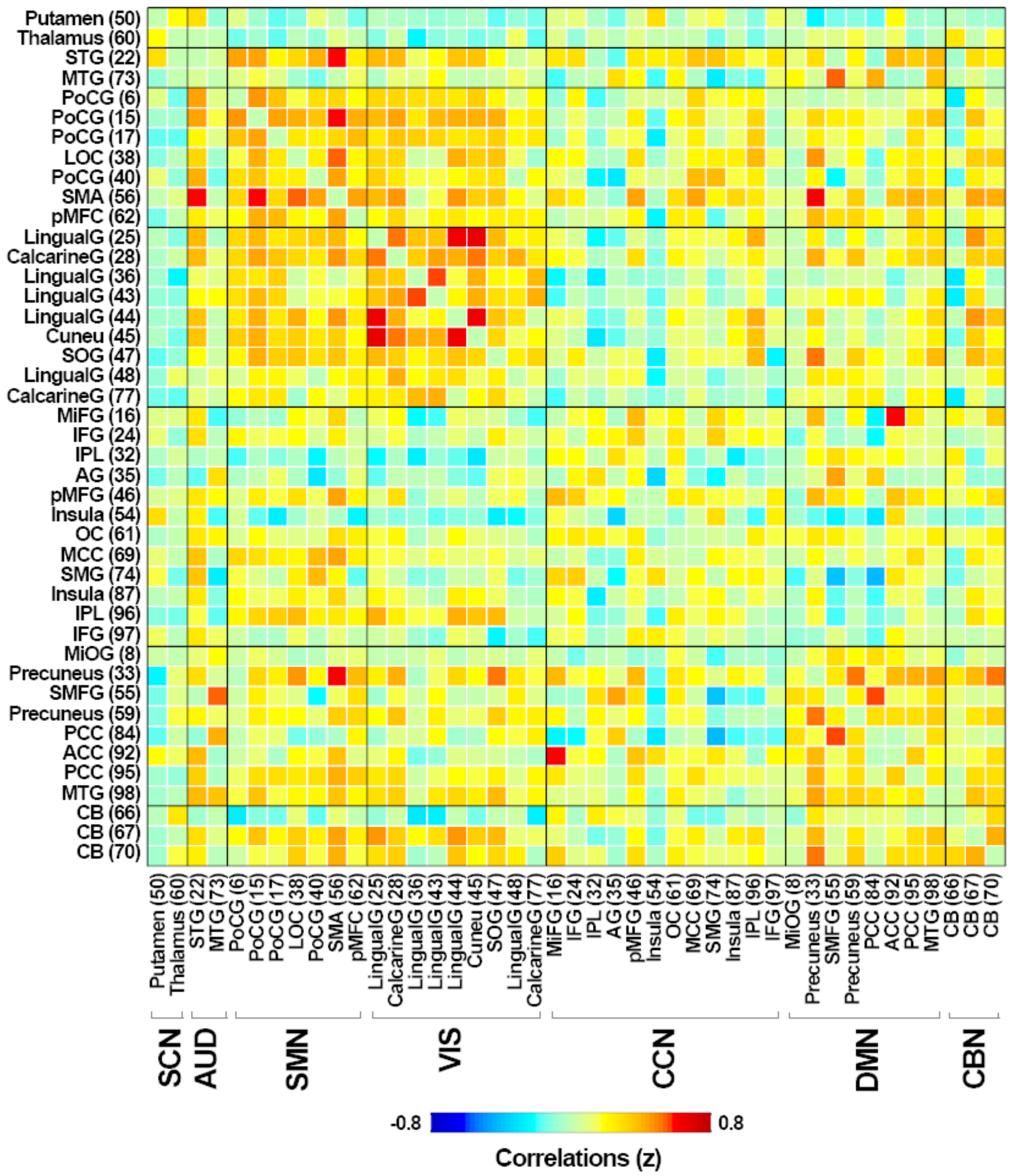

Supplementary Figure 2. Static functional connectivity matrices. The group averaged static functional connectivity matrices were produced using the entire resting state data. Each component was labeled with its corresponding component number.
