# Supplementary material for: Abnormal Static and Dynamic Functional Connectivity in Left and Right Temporal Lobe Epilepsy
Source: Front Neurosci. 2022 Jan 20;15:820641. doi: 10.3389/fnins.2021.820641 (PMC8813030; doi:10.3389/fnins.2021.820641)
Supplement: Supplementary file 3 [file Image_3.pdf]

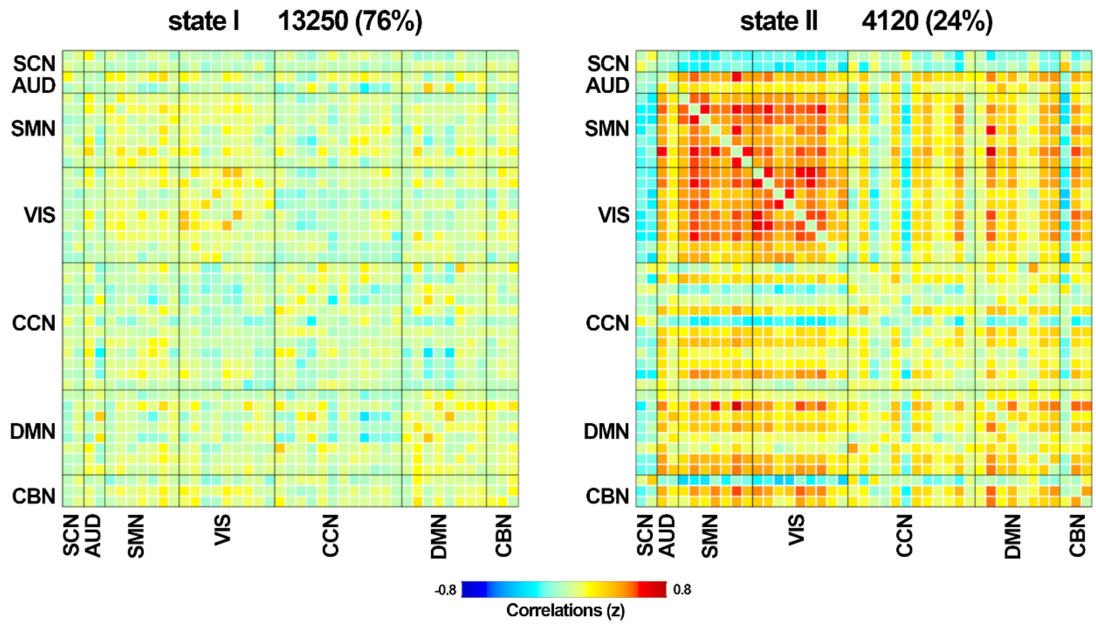

Supplementary Figure 3. The centroids of the clusters for two states that are derived from the k-means clustering algorithm. Each component was labeled with its corresponding component number. State I, which accounted for 76% of all the windows and resembled the static functional connectivity, was characterized by more frequent and weaker connectivity. State II was characterized by less frequent but stronger connectivity.
