# Supplementary material for: Abnormal Static and Dynamic Functional Connectivity in Left and Right Temporal Lobe Epilepsy
Source: Front Neurosci. 2022 Jan 20;15:820641. doi: 10.3389/fnins.2021.820641 (PMC8813030; doi:10.3389/fnins.2021.820641)
Supplement: Supplementary file 4 [file Table_1.pdf]

Supplementary Table 1. Peak Coordinates of Intrinsic Connectivity Networks (ICNs).

ICNs	BA	T-value	MNI coordinate		
			x	y	z
**Subcortical network**					
Putamen (50)	putamen	21.87	26.5	5.5	-3.5
Thalamus (60)	thalamus	34.72	-2.5	-18.5	6.5
**Auditory network**					
STG (22)	superior temoral gyrus	22.8	-59.5	-2.5	-0.5
MTG (73)	middle temporal gyrus	19.93	-56.5	-11.5	-9.5
**Somatomotor network**					
PoCG (6)	postcentral gyrus	19.44	54.5	-6.5	26.5
PoCG (15)	postcentral gyrus	20.06	39.5	-27.5	48.5
PoCG (17)	postcentral gyrus	19.34	-41.5	-30.5	45.5
LOC (38)	lateral occipital cortex	20.89	-15.5	-71.5	59.5
PoCG (40)	postcentral gyrus	24.06	-2.5	-42.5	59.5
SMA (56)	supplementary motor area	24.65	-3.5	-6.5	65.5
pMFC (62)	posterior medial frontal cortex	29.31	3.5	-26.5	59.5
**Visual network**					
LingualG (25)	lingual gyrus	27.65	14.5	-68.5	-9.5
CalcarineG (28)	calcarine gyrus	24.53	9.5	-69.5	11.5
LingualG (36)	lingual gyrus	24.79	-15.5	-53.5	-0.5
LingualG (43)	lingual gyrus	31.44	17.5	-48.5	-2.5
LingualG (44)	lingual gyrus	19.2	-29.5	-81.5	-14.5
Cuneu (45)	cuneus	27	-0.5	-78.5	35.5
SOG (47)	superior occipital gyrus	26.64	27.5	-68.5	42.5
LingualG (48)	lingual gyrus	25.71	5.5	-83.5	-2.5
CalcarineG (77)	calcarine gyrus	27.62	9.5	-57.5	15.5
**Cognitive control network**					
MiFG (16)	middle frontal gyrus	20.96	-30.5	56.5	9.5
IFG (24)	inferior frontal gyrus	24.57	-45.5	14.5	24.5
IPL (32)	inferior parietal lobule	26.21	53.5	-50.5	38.5
AG (35)	angular gyrus	25.13	-47.5	-60.5	33.5
pMFG (46)	posterior medial frontal	24.48	-0.5	11.5	53.5
Insula (54)	insula lobe	27.5	42.5	8.5	-6.5
OC (61)	orbitalis cortex	22.96	45.5	32.5	-17.5
MCC (69)	middle cingulate cortex	33.08	-0.5	3.5	39.5
SMG (74)	supramarginal gyrus	23.95	-60.5	-29.5	33.5
Insula (87)	insula lobe	24.55	-42.5	0.5	-5.5
IPL (96)	inferior parietal lobule	18.13	35.5	-42.5	44.5
IFG (97)	inferior frontal gyrus	20.73	-44.5	21.5	-11.5
**Default mode network**					
MiOG (8)	mid orbital gyrus	28.95	-2.5	45.5	-9.5
Precuneus (33)	precuneus	26.94	-5.5	-65.5	54.5
SMFG (55)	superior medial gyrus	22.83	-0.5	54.5	12.5

ICNs	BA	T-value	MNI coordinate		
			x	y	z
Precuneus (59)	precuneus	28.74	-3.5	-68.5	36.5
PCC (84)	posterior cingulate cortex	36.1	-0.5	-48.5	24.5
ACC (92)	anterior cingulate cortex	24.23	-2.5	24.5	21.5
PCC (95)	posterior cingulate cortex	36.4	-3.5	-20.5	42.5
MTG (98)	middle temporal gyrus	20.15	50.5	-54.5	6.5
**Cerebellar network**					
CB (66)	cerebellum	22.56	18.5	-75.5	-41.5
CB (67)	cerebellum	27.1	27.5	-47.5	-20.5
CB (70)	cerebellum	22.27	-20.5	-68.5	-17.5
